# Munchausen syndrome with factitious hypoglycemia due to deliberate insulin analog administration and factitious hyperglycemia in a patient with hypothyroidism

**DOI:** 10.1186/s40842-022-00145-y

**Published:** 2022-11-15

**Authors:** Marina Yukina, Ilana Katsobashvili, Nadezhda Platonova, Ekaterina Troshina, Galina Mel’nichenko

**Affiliations:** Endocrinology Research Centre, Dm. Ulyanova Street, 11, 117036 Moscow, Russia

**Keywords:** Factitious hyperglycemia, Factitious hypoglycemia, Insulin analogs, Munchausen syndrome

## Abstract

**Background:**

Hypoglycemic syndrome is a potentially life-threatening condition that can lead to the disruption of brain and internal organ functions, and in severe cases to irreparable consequences or death. Factitious hypoglycemia (FH) is the deliberate use of insulin preparations or oral hypoglycemic drugs with the aim of lowering blood glucose levels into the pathologically-hypoglycemic range. Deliberate administration of insulin analogs may be difficult to prove because they might not have epitopes or containing low affinity epitopes that are the targets of antibodies used in particular assay kits.

**Case presentation:**

A 34 years old woman was admitted to the Endocrinology Research Centre in September 2021 with a diagnosis of hypothyroidism and diabetes mellitus. Upon admission she complained of high glycemia indexes up to a maximum of 34 mmol/l ( 612 mg/dl), high TSH and low free T4 ( fT4) concentrations, despite reporting regular levothyroxine administration at a dose of 200 mcg per day. Under nursing supervision, her fT4 was rapidly normalized suggesting non-compliance as the cause of low thyroid hormone milieu. Glycemic fluctuations from 33 to 2.1 mmol/l (594 to 38 mg/dl) according to glucometer measurements were observed against the background of Lis-Pro insulin therapy, while no hyperglycemia was registered in venous blood and in the interstitial fluid concomitantly with the values found by glucometer. It was assumed that the patient’s fingers were intentionally contaminated with glucose solution. Factitious hypo- and hyperglycemia were suspected. During yet another episode of hypoglycemia (1.86 mmol/L, 33 mg/dl) venous blood was drawn. Low to low-normal insulin and C-peptide values were found: 2.2 µU/ml (Roche kit) and 1.18 ng/ml, respectively. Therefore, insulin concentration in the same sample was re-tested with another kit (Abbott) and a significantly elevated value of 89.9 µU/ml was detected. Based on these results, FH was confirmed due to exogenous administration of an insulin analog undetectable by the Roche kit.

**Conclusion:**

This clinical example illustrates to draw attention to multiple manipulations employed by subjects with Munchhausen Syndrome. In addition, this diagnosis may be further complicated by the laboratory use of immunoassay kits incapable of detecting some insulin analogs.

## Background

Factitious hypoglycemia (FH) is the deliberate use of insulin preparations or oral hypoglycemic drugs to reduce blood glucose level [1]. FH is a variant of Munchausen Syndrome [2].

Hypoglycemia (glucose < 55 mg/dL [3.0 mmol/L]) is rare in people without diabetes mellitus (DM) who do not receive hypoglycemic medications due to the good physiological efficiency of counterregulatory mechanisms [3–5].

In the diagnosis of nondiabetic hypoglycemia (NDH), it is always necessary to consider the possibility of FH [5]. The prevalence of FH among other causes of NDH is approximately 10.8% [1]. This condition is the most common in persons with medical education and/or access to diabetes medications [1, 6, 7]. In patients with mental health problems, FH can significantly affect the clinical picture of the disease and the establishment of a correct diagnosis [1, 8].

In the differential diagnosis of the causes of NDH the following are considered: endogenous hyperinsulinism due to insulinoma, autonomous β-cell hypersecretion, autoimmune disorders, status post bariatric surgery, hypoinsulinemic hypoglycemia in hypocorticism and tumors producing insulin-like growth factor 2. In addition, genetically determined disorders of glucose metabolism and insulin secretion can be diagnosed in adult patients in rare cases [1, 5, 9]. FH is one of the most difficult conditions to diagnose [3, 10, 11].

Timely detection of FH avoids costly and time-consuming investigations required to rule out alternative causes of hypoglycemic syndrome [2, 3].

When human insulin preparations are administered, unlike the situation of endogenous hyperinsulinism, C-peptide levels are suppressed because exogenous insulin does not contain C-peptide (as well as proinsulin). However, the determination of recombinant insulins (insulin analogs) can be difficult in laboratories that use kits cross-reacting exclusively with certain epitopes of insulin molecule. In such cases, it might be wrongly assumed that not only C-peptide but also insulin is suppressed [5, 7].

We present the clinical case of Munchausen syndrome in a patient with factitious diabetes mellitus and unresponsiveness to levothyroxine, as well as factitious hypoglycemic events due to insulin analogs administration.

## Case presentation

A 34 years old woman was admitted to the Endocrinology Research Centre (ERC) in September 2021 with a diagnosis of hypothyroidism unresponsive to exogenous levothyroxine as well as exquisetely diabetes mellitus (DM). On admission she complained of impaired swallowing, sore throat, “twitching” pain in the neck, hoarseness of voice, episodic headaches, periorbital swelling mainly in the morning, high glycemia indexes up to a maximum of 34 mmol/l, numbness and lower leg edema.

On examination, her general condition was relatively satisfactory, she was oriented both in place and in time and contact was easy. Her general appearance was unkempt, her clothes and hair were dirty. Her body weight was 95 kg, height 166 cm, body mass index 35.2 kg/m.sq. On her back there were areas of vitiligo, the skin of the front surfaces of both thighs contained rough scars (according to the patient, post-injection abscesses), and the rest of her physical examination was noncontributory.

She had three children, was a very heavy smoker and alcohol drinker. She was not employed despite an engineering degree. She had paternal relative with diabetes mellitus (DM) of unknown type.

According to the patient, in November 2020, hypothyroidism was diagnosed based on the results of hormonal blood tests (data not available), and she was prescribed levothyroxine sodium with a gradual titration of the daily dose to 200 mcg, i.e. 2.1 mcg/kg.

In March 2021, there was an episode of unconsciousness, an ambulance team was called, and she was hospitalized with hyperglycemia of 26 mmol/l (468 mg/dl). Intensive insulin therapy (Insulin lispro 8–35 units 3 times a day and Insulin-isophan 30 units in the morning and in the evening) was initiated at the hospital. After discharge, due to complaints of high glycemia indices, Metformin 1000 mg twice daily was added, but hyperglycemias up to 34 mmol/l ( 612 mg/dl) still occurred on glucometer self-measurements. She was therefore referred to ERC for thorough work-up and treatment.

On September 21, 2021, she was hospitalized for the first time at the ERC. Decompensation of primary hypothyroidism against the background of autoimmune thyroiditis was confirmed: TSH 108 mIU/l, AT to TPO > 1000 mU/ml, AT to TG > 40,000 mU/ml, fT4 < 5.15 pmol/l (0.4 ng/dl; reference interval 0.69–1.47 ng/dl). The dose of levothyroxine was increased to 300 mcg and given under nursing supervision and fT4 level was normalized at 11.29 pmol/l ( 0.88 ng/dl) after 5 days, strongly suggesting non-compliance at home.

In the hospital, she had widely fluctuating glycemia indices from 33 mmol/l (594 mg/dl) to 2.1 mmol/l (37 mg/dl) during standard diabetic diet and her home insulin regimen. However, no hyperglycemia was registered in venous blood even during simultaneous very high blood glucose levels according to the glucometer measurements. She never had positive urinary ketones.

On September 22, 2021, she was transferred to the intensive care unit due to a sudden episode of mental confusion, accompanied by nausea, vomiting, headache, with a background glycemia of 3.2 mmol/L (58 mg/dl). In the intensive care unit, under strict nursing supervision, glycemic readings were all normal within 4.5–8.3 mmol/l (81–159 mg/dl) for 2 days despite complete withdrawal of both insulin therapy and metformin, but with slow intravenous infusion of 5% glucose solution. An oral glucose tolerance test (OGTT) was performed and no evidence of DM was obtained: fasting glucose was 4.96 mmol/l (90 mg/dl), and 2 h later it was 7.39 mmol/l (133 mg/dl). Therefore, artifical hypo- and hyperglycemia was suspected.

Back on the general ward continuous interstitial glycemia monitoring *Medtronic Paradigm Vео MMT-754*) was initiated. Elevated glucose values (up to 18 mmol/L, 324 mg/dl) were recorded only by glucometer, while no hyperglycemia was detected in the interstitial fluid using glucose sensor at the same time. Very low glucose values were repeatedly noted, down to 2.1 mmol/l (38 mg/dl), and during one such episode venous blood sampling was performed: glucose 1.86 mmol/l ( 33 mg/dl), insulin 2.2 µU/ml (Roche), C-peptide 1.18 ng/ml and proinsulin less than 0.5 pmol/l. Presence of oral glucose-lowering medications in the blood was excluded by High performance liquid chromatography coupled with tandem mass spectrometry (HPLC-MS/MS). Taking into account the discordant values of insulin and C-peptide it was decided to perform a fasting test to re-examine these hormones during hypoglycemia.

She developed clinical hypoglycemia (accompanied by severe weakness, excessive sweating, trembling) after 1.5 h of fasting; venous blood samples were taken (Table 1) and fasting was stopped (the patient ate).

In addition, laboratory indicators were also suspicious of factitious hypoglycemia due to the introduction of insulin analogues, so it was decided to conduct an additional study of insulin with the Abbot kit.

**Table 1:** The Patient’s Laboratory Data at the end of the Fasting Test

Test	Value (units)	Reference interval
Glucose	1.64 mmol/l	3.1–6.1
Insulin (RE)*	3.16 µU/ml	2.6-24.9^1^
Insulin (AA) **	89.9 µU/ml	2.6-24.9^1^
С-peptide	1.41 ng/ml	1.1–4.4^1^
beta-hydroxybutyrate	< 2.7 mmol/l	> 2.7

* kit Roche

** kit Abbott

^1^—Reference interval for euglycemia

The feature of these results was very low blood glucose level accompanied by low-normal insulin concentration in the Roche’s assay but with grossly elevated value in the Abbott’s assay. Thus, the presence of artificial hypoglycemia due to exogenous administration of insulin analog preparation, i.e. the diagnosis of Munchausen syndrome, was confirmed. Taking into account the patient’s customary home use of ultra-short-acting insulin (insulin lispro) we assume that this drug was surreptitiously administered by the patient to induce hypoglycemic episodes. Unfortunately, at that time our medical institution did not have an opportunity to identify the insulin preparations by HPLC-MS/MS.

After discussing the results with the patient, we told her that continuation of surreptitious insulin injections to induce hypoglycemia puts her life and health in danger. However, the patient calmly denied surreptitious administration of insulin preparations. A psychiatrist consulted the patient and diagnosed her with chronic anxiety-depressive disorder with panic attacks. The patient was given recommendations for drug treatment and psychotherapy.

## Discussion

This patient presented with some features suspicious of a psychiatric disorder and malingering. Persistently elevated TSH and low fT4 despite the above-replacement doses of levothyroxine were due to noncompliance. When she was admitted to the intensive care unit with 24-hour nursing observation and, accordingly, had no access to personal belongings, euglycemia was observed during the entire 2-day period despite omission of insulin and metformin and her hypoglycemic episodes ceased to exist. Subsequent oral glucose loading test firmly excluded the very diagnosis of diabetes mellitus.

The installation of a continuous glucose monitoring device made it possible to see a discrepancy between the high blood glucose reading from the fingertip and normal glucose values in the interstitial fluid and/or venous blood. Most likely, she was intentionally contaminated the skin of her fingertips with glucose solution and letting it dry before the blood draw for glucometer measurement.

However, the most important feature of this case was the finding of falsely low blood insulin levels during hypoglycemic episodes. When human insulin is administered, insulin levels increase while C-peptide and proinsulin levels become suppressed because exogenous insulin lowers glucose levels and aborts proinsulin, insulin and C- peptide secretion from the beta cells. However, in the presented case insulin, C-peptide and proinsulin were all low in the presence of hypoglycemia, suggesting hypoinsulinemic hypoglycemia and seemingly excluding surreptitious insulin administration. Only realization that insulin assays were done with the Roche’s kit while the subsequent re-testing of the same sample with the Abbott’s kit showed extremely high insulin values were we able to establish a correct diagnosis.

Human insulin contains two polypeptide chains that are 21 (A-chain) and 30 (B-chain) aminoacid residues in length. Lis-pro analog used for the treatment of DM has aminoacid sequence modifications in the C-terminal part of the B-chain. Therefore, since Roche assay is specifically designed to detect endogenous human insulin by targeting the intact carboxy end of the B-chain it has low cross-reactivity with Lis-pro insulin analog, whereas other assays kits target different epitopes and therefore are capable to detect even modified insulin molecules, including Lis-Pro [12].

In 2001 Sapin et al. [13] first suggested the absence of cross-reactivity with insulin analogues in some assays [13], which was subsequently confirmed by other groups [12, 14–16]. There are also a number of publications with clinical cases on this topic [17–20]. Chemmanam et al. [20] reported two cases of hypoglycemia where the diagnosis was initially unclear due to the use of the RE assay. In both cases insulin concentration was normal when measured with the Roche’s immunoassay, but was appropriately elevated when remeasured with the Advia Centaur immunoassay and a diagnosis of hypoglycemia secondary to insulin analogue administration was made. In addition, this work compared 4 analytical platforms with respect to the detection of 5 insulin preparations [20]. These cases emphasize that understanding the binding characteristics of the insulin immunoassay used by the laboratory is essential to making a correct diagnosis.

Thus, laboratory services play a key role in the differential diagnosis of exogenous administration of insulin analogues and physicians must be made aware of the specificity of the kit used to interpret the correct results.

In addition, another feature of the case is borderline ambiguous results of hormonal parameters against the background of hypoglycemia.

In this case there was no complete suppression of C-peptide (to less than 0.6 ng/ml) and insulin levels were still detectable at around 3 µU/ml or less, but both values were borderline-low. Potentially, this might be due a longer half-life of C-peptide compared to insulin [21], and possibly to the presence of markedly elevated insulin levels due to insulin resistance in a patient with obesity. Thus, getting borderline results of insulin and C-peptide during the background of hypoglycemia should be interpreted very carefully.

## Conclusion

Thus, the enigma of widely fluctuating glycemia, from elevated to low levels was attributable to surreptitious manipulations by the patient as well as to the laboratory peculiarity. Once again, it emphasizes the complexity of the diagnosis of Munchausen Syndrome that becomes even more difficult in the era of multiple biosynthetic insulin preparations and a multitude of immunoassays with different binding properties.

## Data Availability

All data generated or analysed during this study are included in this published article.

## Abbreviations

FH: Factitious Hypoglycemia
NDH: Nondiabetic hypoglycemia
fT4: Free thyroxine
HPLC-MS/MS: High performance liquid chromatography coupled with tandem mass spectrometry
DM: Diabetes mellitus
ERC: Endocrinology Research Centre
OGTT: Oral glucose tolerance test
RE: ROCHE ELECSYS
AA: ABBOTT ARCHITECT
